# Effect of dialysis modalities on risk of hospitalization for gastrointestinal bleeding

**DOI:** 10.1038/s41598-022-26476-5

**Published:** 2023-01-02

**Authors:** Chieh-Hsin Huang, Jo-Yen Chao, Tsai-Chieh Ling, Jia-Ling Wu, Junne-Ming Sung, Chien-Yao Sun, Ya-Yun Cheng, Yu-Tzu Chang

**Affiliations:** 1 Department of Internal Medicine, National Cheng Kung University Hospital, College of Medicine, National Cheng Kung University, 138 Sheng-Li Rd., Tainan, 70428 Taiwan; 2 Department of Public Health, College of Medicine, National Cheng Kung University, Tainan, Taiwan; 3 Department of Geriatrics and Gerontology, National Cheng Kung University Hospital, College of Medicine, National Cheng Kung University, Tainan, Taiwan; 4 Department of Post-Baccalaureate Medicine, College of Medicine, National Sun Yat-Sen University, Kaohsiung, Taiwan

**Keywords:** Renal replacement therapy, Gastrointestinal bleeding

## Abstract

Dialysis patients are at risk of both thromboembolic and bleeding events, while thromboembolism prevention and treatment may confer a risk of major bleeding. Gastrointestinal (GI) bleeding is a great concern which can result in high subsequent mortality rates. Our object was to clarify whether hemodialysis (HD) and peritoneal dialysis (PD) confer different incidence of GI bleeding, and further assist individualized decision-making on dialysis modalities. We conducted a population-based retrospective cohort study which included all incident dialysis patients above 18 years old derived from the National Health Insurance database from 1998 to 2013 in Taiwan. 6296 matched pairs of HD and PD patients were identified. A propensity score matching method was used to minimize the selection bias. The adjusted hazard ratio for GI bleeding was 1.13 times higher in the HD group than in the PD group, and data from the unmatched cohort and the stratified analysis led to similar results. Among subgroup analysis, we found that the use of anticoagulants will induce a much higher incidence of GI bleeding in HD patients as compared to in PD patients. We concluded that PD is associated with a lower GI bleeding risk than HD, and is especially preferred when anticoagulation is needed.

## Introduction

The incidence and prevalence of kidney failure requiring maintenance dialysis has grown rapidly worldwide in recent decades^1^. According to an annual report from the United States Renal Data System (USRDS), the incidence of kidney failure varies greatly across countries, and Taiwan has typically ranked the highest in the world. The enormous healthcare expenditures required for long term dialysis therapy^2,3^, dialysis-related comorbidities and mortalities, and the low employment rates^4^ of dialysis patients will inevitably result in a high financial and healthcare burden^5^.

Patients with kidney failure are at higher risk of thromboembolic events^6^ and are especially prone to developing ischemic stroke when atrial fibrillation (Af) is present^7^. Thus, anticoagulation therapy for primary or secondary thromboembolic prevention is often considered^8,9^. Meanwhile, the incidence of bleeding events is known to be high in dialysis patients^10–12^. Determining how to create a balance between the benefit of anticoagulants and the bleeding risk has always been a challenge for clinical physicians. Among these major bleeding events, gastrointestinal (GI) bleeding is a common, but frequently underestimated medical condition that can result in subsequent morbidity and mortality. It is reported that patients with GI bleeding have comparable mortality rates to those with acute myocardial infarctions (AMI)^13^, and GI bleeding-related mortality has been shown to be even higher among patients with kidney failure^14^. In addition, patients on long-term dialysis have a higher rebleeding risk than non-dialysis controls after peptic ulcer bleeding (PUB)^15^. To ameliorate the high mortality in the dialysis population, the potential risk of GI bleeding should be carefully assessed in clinical practice.

It is already known that patients on hemodialysis (HD) have a greater risk of GI bleeding as compared to their matched controls or the general population^16–18^. An observational cohort study showed that the incidence of bleeding events was 60.8 and 34.6 per 1000 person-years for HD and peritoneal dialysis (PD) patients, contrasting with the general population of 0.5–0.9 per 1000 person-years^19^. It is thus suggested that HD patients generally have a higher bleeding risk than those on PD. However, whether dialysis modalities per se confer a differential risk for GI bleeding remains to be clarified^11,20,21^. Therefore, we performed a nationwide population-based cohort study to quantify the differential risk for GI bleeding in patients treated with HD and PD using the National Health Insurance Research Database (NHIRD) in Taiwan.

## Methods

### Data source

We applied data derived from the medical information of all beneficiaries from the Taiwan’s National Health Insurance (NHI) program. It is a nationwide health insurance system launched in 1995 that reimburses almost all medical services, including inpatient/outpatient services, procedures, prescriptions, and health education. The system is mandatory for all citizens, and the copayments for medical services are low. Therefore, the coverage rate of the NHI program is as high as 99% of the entire population in Taiwan. To ensure personal privacy while providing public health information for research purposes, all personal information is encrypted before releasing. Thus, this study was approved by the Institutional Review Board (IRB) of the National Cheng Kung University Hospital (B-EX-108-024) and the need for informed consent was also waived by it. All research procedures performed in this study were in accordance with the ethical standards of the institutional and national research committee and also with the 1964 Helsinki declaration and its later amendments or comparable ethical standards.

According to the NHI regulations, patients receiving maintenance dialysis can be certified as having experienced a catastrophic illness and can be exempted from copayments for medical services. Information related to kidney failure, including the associated etiologies, indications, images and laboratory data, are carefully reviewed by experts during the certification process. Therefore, the accuracy of the diagnosis of kidney failure is ensured.

### Identification of the study population

**Figure 1:**
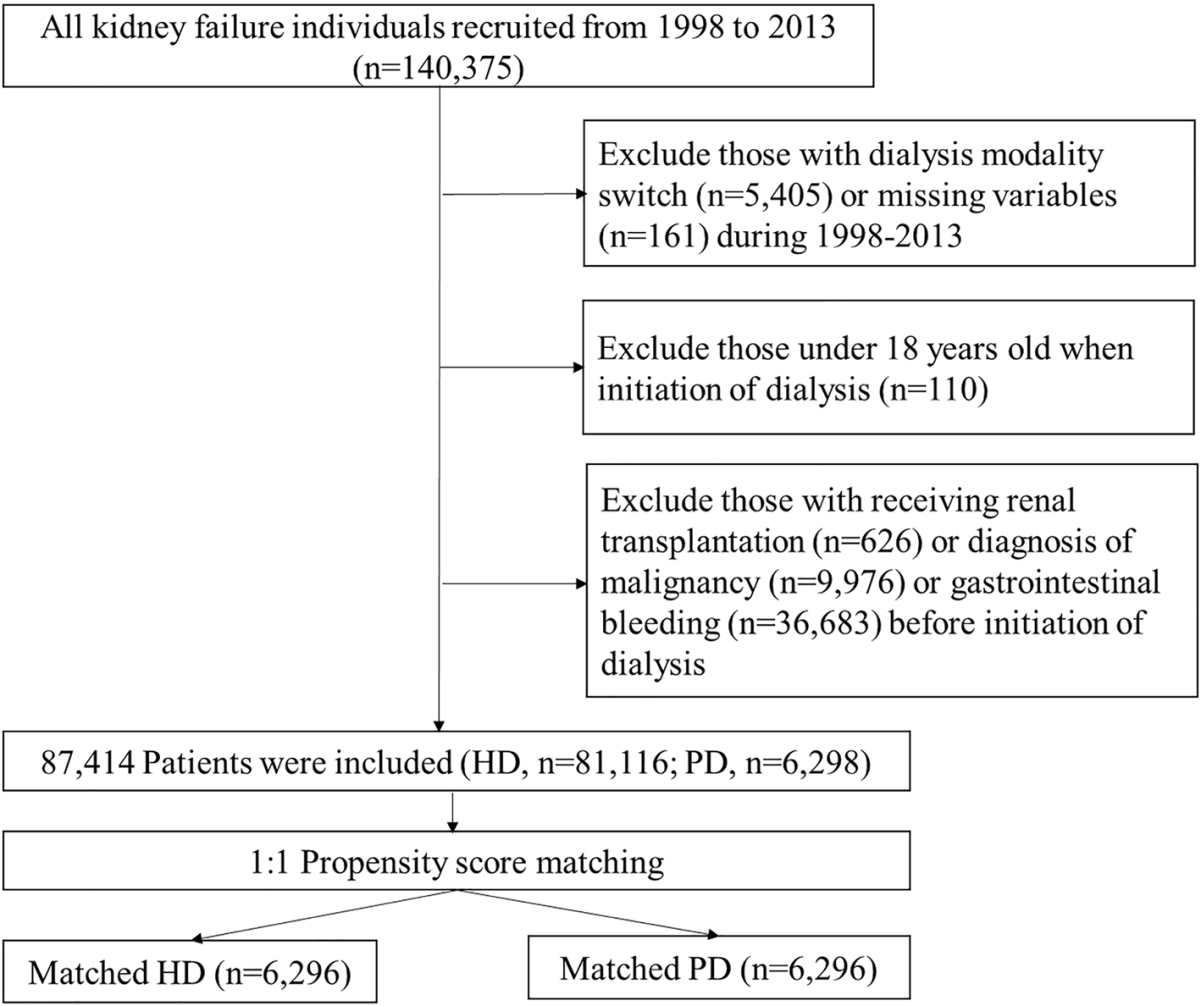
Flow chart of the establishment of matched pairs of patients receiving hemodialysis (HD) and peritoneal dialysis (PD) from the national cohort of kidney failure patients. Abbreviation: HD: hemodialysis, PD: peritoneal dialysis.

To compare the risk differences in incident GI bleeding between patients receiving HD and PD, we conducted a population-based retrospective propensity score-matched cohort study. We first identified all patients with incident kidney failure initiating maintenance dialysis for more than three consecutive months between January 1, 1998, and December 31, 2013. Exclusion criteria included: age < 18 years old at the initiation of dialysis, had received a renal transplantation, had a diagnosis of GI bleeding or malignancy before the initiation of dialysis, had switched dialysis modalities for three consecutive months during the follow-up period, or missing variables at the time of enrollment (Fig. 1).

### Outcome measures and identification of research variables

The primary outcome of this study was the incidence of hospitalization for all types of GI bleeding, defined by the International Classification of Diseases, 9th Revision (ICD-9) codes, during the follow-up period (Supplementary Table S1). The date of the outcome occurrence was recorded as the first date of the hospitalization for GI bleeding. The subtypes of GI bleeding, including upper, lower, and unspecified GI bleeding, were also identified for a further analysis. To reduce potential confounding effects, we obtained information related to several comorbidities and medications listed in Table 1 and Supplementary Tables S2, S3, which have been proven to be related to the risk of GI bleeding^22–25^. The selected comorbidities and medications were defined by using the ICD-9 and Anatomical Therapeutic Chemical codes, respectively, as listed in Supplementary Tables S2 and S3. Patients were defined as having these comorbidities if they were diagnosed once in the inpatient claim data or at least twice in the outpatient claim data with ≥ 30 days apart in one year prior to the initiation of dialysis. Patients prescribed with the medication listed in Table 1 within one year before the initiation of dialysis were defined as a user of these medications. Patients were censored at the date of mortality, after receiving kidney transplantation, after withdrawal from the NHI program, or at the end of the study period, December 31, 2013.

**Table 1:** Comparison of the demographic and clinical characteristics of dialysis patients before and after matching using propensity scores.

	Before matching			After matching		
	All HD patients	All PD patients	*p* value	Matched HD patients	Matched PD patients	di
Number of patients	81,116	6298		6296	6296	
Age, mean (SD)	61.51 (14.06)	53.36 (15.03)	< 0.0001	52.89 (15.04)	53.37 (15.03)	3.24
**Age group, no. (%)**						
18–34 years	3081 (3.80)	747 (11.86)	< 0.0001	816 (12.96)	745 (11.83)	3.42
35–49 years	13,150 (16.21)	1705 (27.07)		1726 (27.41)	1705 (27.08)	0.75
50–64 years	28,704 (35.39)	2405 (38.19)		2301 (36.55)	2405 (38.2)	3.41
65–79 years	28,574 (35.23)	1157 (18.37)		1213 (19.27)	1157 (18.38)	2.28
≥ 80 years	7607 (9.38)	284 (4.51)		240 (3.81)	284 (4.51)	3.50
**Sex, no. of male (%)**	41,310 (50.93)	3006 (47.73)	< 0.0001	3020 (47.97)	3006 (47.74)	0.45
**Index year**			< 0.0001			
1998–2001	18,400 (22.68)	125 (1.98)		155 (2.46)	125 (1.99)	3.23
2002–2005	18,781 (23.15)	368 (5.84)		392 (6.23)	368 (5.84)	1.60
2006–2009	21,093 (26.00)	2691 (42.73)		2750 (43.68)	2691 (42.74)	1.89
2010–2013	22,842 (28.16)	3114 (49.44)		2999 (47.63)	3112 (49.43)	3.59
Duration of follow-up, years	3.96 (3.63)	2.99 (2.32)	< 0.0001	3.41 (2.67)	2.99 (2.32)	16.88
**Comorbidities, no. (%)**						
Diabetes mellitus	46,571 (57.41)	2496 (39.63)	< 0.0001	2453 (38.96)	2496 (39.64)	1.40
Hypertension	66,960 (82.55)	5072 (80.53)	< 0.0001	5054 (80.27)	5070 (80.53)	0.64
Coronary artery disease	27,645 (34.08)	1463 (23.23)	< 0.0001	1406 (22.33)	1463 (23.24)	2.16
Peripheral vascular disease	6647 (8.19)	359 (5.7)	< 0.0001	311 (4.94)	359 (5.70)	3.40
Heart failure	25,895 (31.92)	1276 (20.26)	< 0.0001	1265 (20.09)	1276 (20.27)	0.44
Stroke	12,349 (15.22)	539 (8.56)	< 0.0001	528 (8.39)	539 (8.56)	0.63
Chronic obstructive pulmonary disease	13,984 (17.24)	690 (10.96)	< 0.0001	615 (9.77)	690 (10.96)	3.91
Hyperlipidemia	30,513 (37.62)	2626 (41.7)	< 0.0001	2548 (40.47)	2624 (41.68)	2.45
Rheumatological diseases	2855 (3.52)	331 (5.26)	< 0.0001	301 (4.78)	331 (5.26)	2.18
**Baseline medication, no. (%)**						
Aspirin	25,507 (31.45)	1279 (20.31)	< 0.0001	1286 (20.43)	1279 (20.31)	0.28
Antiplatelet agent	11,194 (13.8)	678 (10.77)	< 0.0001	659 (10.47)	678 (10.77)	0.98
Non-steroid anti-inflammation drug	47,513 (58.57)	3073 (48.79)	< 0.0001	3063 (48.65)	3071 (48.78)	0.25
Cox-2 selective inhibitors	4248 (5.24)	237 (3.76)	< 0.0001	217 (3.45)	237 (3.76)	1.70
Corticosteroids	19,327 (23.83)	1595 (25.33)	0.0072	1514 (24.05)	1593 (25.3)	2.91
Selective serotonin reuptake inhibitors	2409 (2.97)	143 (2.27)	0.0015	125 (1.99)	143 (2.27)	1.98
Anticoagulants	1807 (2.23)	94 (1.49)	0.0001	97 (1.54)	94 (1.49)	0.39
Gastroprotective agents	27,472 (33.87)	1981 (31.45)	< 0.0001	1908 (30.3)	1981 (31.46)	2.51
Aldosterone antagonists	9,199 (11.34)	461 (7.32)	< 0.0001	446 (7.08)	461 (7.32)	0.92
Calcium channel blocker	65,926 (81.27)	5182 (82.28)	0.0483	5160 (81.96)	5180 (82.27)	0.83
Nitrates	29,311 (36.13)	1396 (22.17)	< 0.0001	1381 (21.93)	1396 (22.17)	0.57

*HD* Hemodialysis; *PD* Peritoneal dialysis; *di*, Standardized differences; *SD:* Standard deviation.

### Construction of the logistic models to estimate the propensity scores for matching

To reduce potential selection bias due to dialysis modality, we used a logistic regression model to estimate the propensity score, which represented an unbiased estimate of all confounders regarding dialysis modalities. The independent variables in the logistic regression model included age, sex, index year of initiation of dialysis therapy, selected baseline comorbidities, and the use of the baseline medications listed in the Table 1. These variables were selected because it has been proven that including variables unrelated to exposure but related to the outcome will decrease the variance of an estimated exposure effect without increasing bias^26^. A 1:1 propensity score matching method obtained using the nearest neighbor matching method algorithm was performed to construct the matched pairs of HD and PD patients. The caliper width of the nearest score was set to range between − 0.1 and + 0.1.

### Statistical analysis

Comparisons between the continuous and categorical variables in the unmatched cohort were conducted using a Student’s t-test, a chi-square test, or Fisher's exact tests, as appropriate. The balance of the covariate distribution in the matched cohort was evaluated using the standardized difference. The incidence rates (IRs) for the outcome variables were estimated under the Poisson assumption. To minimize any possible estimation bias caused by the long follow-up period and high mortality rate of the dialysis patients in this study, we adopted a cumulative incidence competing risk analysis and multivariable Cox proportional subdistribution hazard regression models to estimate cumulative incidence rates (CIRs) and adjusted subdistribution hazard ratios (aSHRs)^27,28^. Between-group differences in the CIR for the HD and PD group were evaluated using the modified Gray’s test^29^. Interaction between variables was assessed with the Cox regression model. All statistical tests were performed using SAS version 9.4 (SAS Institute, Cary, NC). A two-sided *p* value < 0.05 was considered statistically significant.

## Results

### Baseline characteristics of patients before and after matching

A total of 87,414 patients were eligible to be enrolled in this study, of which 81,116 and 6298 patients received HD and PD, respectively (Fig. 1). The mean follow-up periods for the HD and PD groups were 3.96 and 2.99 years, respectively. Table 1 presents the baseline characteristics, comorbidities, and concomitant use of medications for these dialysis patients. Among the unmatched cohort, the HD patients were older and had significantly higher prevalence of comorbidities with the exception of hyperlipidemia and rheumatological diseases. HD patients had greater exposure to the use of all selected medications, with the exception of corticosteroid and calcium channel blockers. After performing the propensity score matching, 6296 matched pairs of HD and PD patients were identified. The distribution of the baseline characteristics, selected comorbidities, and medication use were successfully balanced after matching.

### Comparison of GI bleeding risks between the HD and PD groups

**Figure 2:**
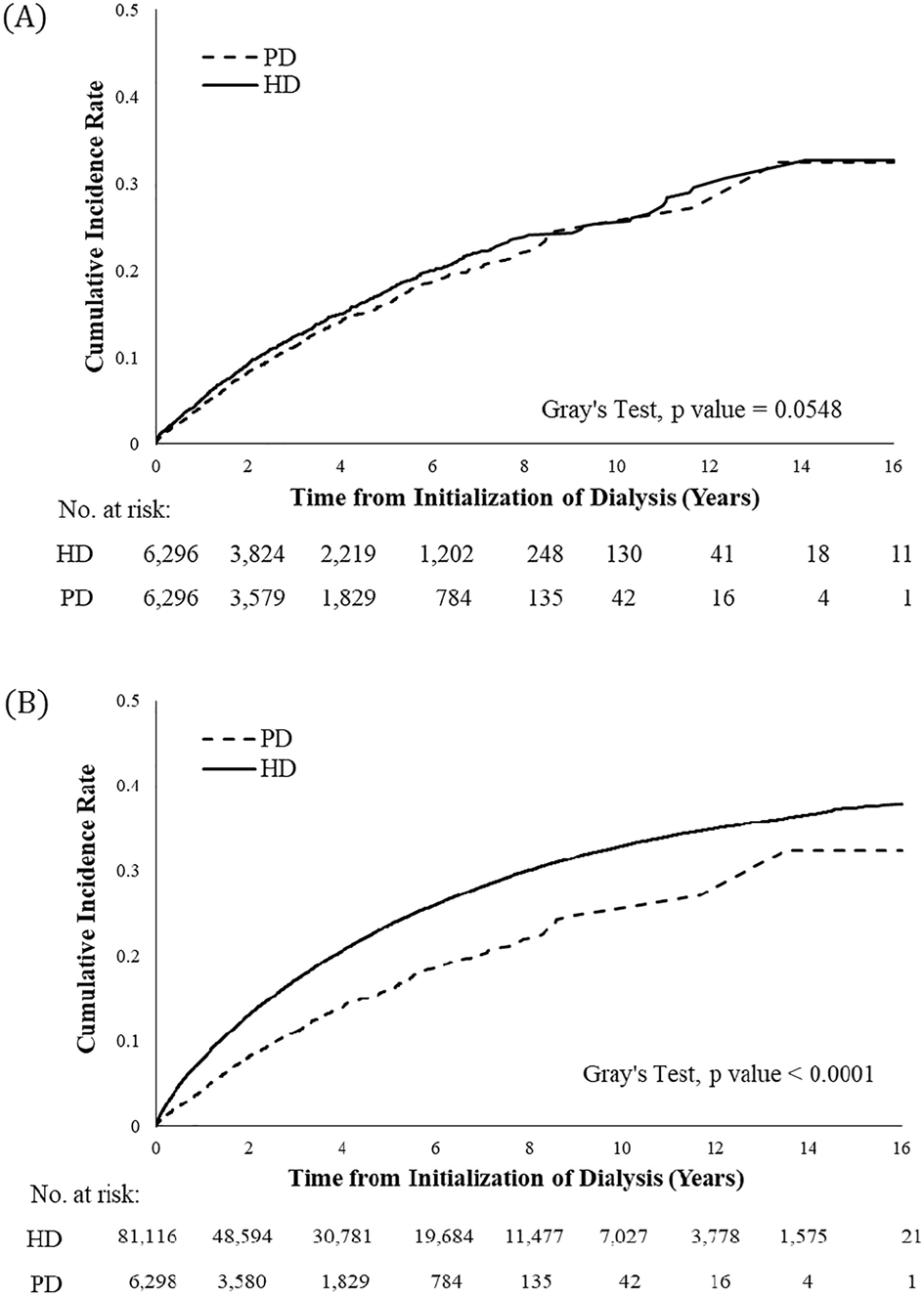
Cumulative incidence rates (CIRs) of gastrointestinal bleeding after accounting for the competing risk of mortality in matched (**A**) and unmatched (**B**) patients with hemodialysis (HD) and peritoneal dialysis (PD).

The IRs of GI bleeding are shown in Table 2. Among the matched cohort, the overall IR of the HD group was slightly higher than that of the PD group (44.23 vs. 43.31 per 1000 patient-years). When stratified by sex and age, the IRs generally increased with age in both groups. However, the IRs of the matched HD patients were not consistently higher than those of the PD patients across every age- and sex-stratification. When considering the difference in the CIRs, the HD patients still exhibited higher rates of GI bleeding compared with the PD patients over the 16-year follow-up period, with borderline significance (0.3265 vs. 0.3240, *p* = 0.05) (Fig. 2).

**Table 2:** Comparison of incidence rates (per 1000 patient-years) and relative risk of gastrointestinal bleeding between patients with hemodialysis (HD) and peritoneal dialysis (PD) before and after matching using propensity scores.

Characteristics	Before matching					After matching				
	All PD patients		All HD patients			Matched PD patients		Matched HD patients		
	No. of events	Incidence rates	No. of events	Incidence rates	aSHR† (95% CI) (Ref. = PD)	No. of events	Incidence rates	No. of events	Incidence rates	aSHR† (95% CI) (Ref. = PD)
Overall	815	43.30 (40.38–46.38)**	20,970	65.25 (64.37–66.13) **	1.23** (1.15–1.33)	815	43.31 (40.39–46.39)	950	44.23 (41.46–47.13)	1.13* (1.03–1.24)
**Male age group**										
18–34	33	30.94 (21.30–43.45)	285	26.21 (23.25–29.43)	0.98 (0.66–1.46)	33	30.94 (21.30–43.45)	42	26.57 (19.15–35.92)	0.87 (0.53–1.43)
35–49	82	32.73 (26.03–40.63)	1584	42.3 (40.25–44.44)	1.26 (1.00–1.60)	82	32.73 (26.03–40.63)	120	39.44 (32.70–47.16)	1.24 (0.93–1.66)
50–64	191	56.92 (49.14–65.59)	3977	66.01 (63.97–68.09)	1.09 (0.94–1.27)	191	56.92 (49.14–65.59)	187	52.28 (45.05–60.33)	1.01 (0.82–1.23)
65–79	88	75.52 (60.57–93.04)	3803	95.43 (92.42–98.51)	1.29* (1.05–1.60)	88	75.52 (60.57–93.04)	104	74.61 (60.96–90.4)	1.09 (0.82–1.47)
≥ 80	19	110.01(66.23–171.79)	878	131.57 (123.01–140.57)	1.45 (0.91–2.31)	19	110.01 (66.23–171.79)	24	131.71 (84.39–195.97)	1.54 (0.76–3.12)
Total	413	49.97(45.27–55.03)**	10,527	67.88 (66.59–69.18)**	1.21** (1.09–1.34)	413	49.97 (45.27–55.03)	477	48.79 (44.51–53.37)	1.10 (0.97–1.26)
**Female age group**										
18–34	26	17.26 (11.28–25.29)	182	20.38 (17.52–23.56)	1.06 (0.65–1.73)	26	17.31 (11.31–25.36)	31	19.49 (13.24–27.66)	0.90 (0.48–1.69)
35–49	78	22.95 (18.14–28.64)	1196	29.64 (27.98–31.37)	1.00 (0.77–1.29)	78	22.95 (18.14–28.64)	78	21.33 (16.86–26.63)	0.91 (0.66–1.26)
50–64	157	40.47 (34.39–47.32)	3452	57.36 (55.46–59.31)	1.06 (0.89–1.25)	157	40.47 (34.39–47.32)	175	41.89 (35.91–48.57)	1.06 (0.85–1.32)
65–79	109	71.91 (59.05–86.75)	4532	92.17 (89.51–94.89)	1.33*(1.10–1.62)	109	71.91 (59.05–86.75)	158	77.9 (66.23–91.04)	1.27 (0.99–1.63)
≥ 80	32	124.8 (85.36–176.18)	1081	140.9 (132.62–149.55)	1.43 (1.00–2.05)	32	124.8 (85.36–176.18)	31	124.8 (84.79–177.14)	1.11 (0.65–1.89)
Total	402	38.08(34.45–41.99)**	10,443	62.79 (61.59–64.01)**	1.24** (1.12–1.38)	402	38.09 (34.46–42.01)	473	40.42 (36.86–44.23)	1.15** (1.00–1.31)

The comparisons of overall and sex-specific incidence rates and cumulative incidence rates between the HD and PD patients without matching were all statistically significant (*p* < 0.0001), and the male and female incidence rates for the HD and PD patients with matching were statistically significant (*p* < 0.05).

*aSHR* Adjusted subdistribution hazard ratio; *Ref.* Reference group; *CI* Confidence interval.

^†^Based on a Cox proportional hazard regression with a competing risk analysis and adjusted for age, sex, selected comorbidities (diabetes mellitus, hypertension, coronary artery disease, peripheral vascular disease, heart failure, stroke, chronic obstructive pulmonary disease, hyperlipidemia, rheumatological disease) and medications (Aspirin, Antiplatelet agent, NSAID, Cox-2 selective inhibitors, Corticosteroids, Selective Serotonin Reuptake Inhibitors, Anticoagulants, Gastroprotective agents, Aldosterone antagonists, Calcium channel blocker, Nitrates).

*p value < 0.05.

**p value < 0.001.

After adjusting for potential confounders using Cox proportional subdistribution hazard models, the HD patients were associated with a higher risk of GI bleeding than the PD patients (aSHR: 1.13, 95% confidence interval [CI]: [1.03–1.24]) (Table 2). Furthermore, we investigated whether the effects of the dialysis modality were consistent when outcomes were categorized into upper, lower, and unspecified GI bleeding groups (Table 3). The results still indicated that the HD patients were associated with a higher, but not statistically significant, risk of developing all subtypes of GI bleeding (aSHR of upper GI 1.11, 95% CI 0.99–1.25; aSHR of lower GI 1.26, 95% CI 0.83–1.91; aSHR of unspecified GI: 1.18, 95% CI 0.99–1.40).

**Table 3:** The event numbers and the estimated subdistribution hazard ratios for different types of GI bleeding using a multivariable regression model.

	Before matching			After matching		
	HD (n = 81,116)	PD (n = 6298)	aSHR (95% CI)	HD (n = 6296)	PD (n = 6296)	aSHR (95% CI)
**Overall bleeding, no. (%)**	20,970 (25.85)	815 (12.94)	1.23** (1.15–1.33)	950 (15.09)	815 (12.94)	1.13* (1.03–1.24)
Upper GI bleeding	14,150 (17.44)	567 (9.00)	1.23** (1.12–1.34)	653 (10.37)	567 (9.01)	1.11 (0.99–1.25)
Lower GI bleeding	1231 (1.52)	40 (0.64)	1.49* (1.08–2.06)	51 (0.81)	40 (0.64)	1.26 (0.83–1.91)
Unspecified bleeding	5589 (6.89)	208 (3.30)	1.24* (1.09–1.42)	246 (3.90)	208 (3.30)	1.18 (0.99–1.40)

*aSHR* Adjusted subdistribution hazard ratio; *CI* Confidence interval.

aHRs were adjusted for age, sex, selected comorbidities (diabetes mellitus, hypertension, coronary artery disease, peripheral vascular disease, heart failure, stroke, chronic obstructive pulmonary disease, hyperlipidemia, rheumatological disease) and medications (Aspirin, Antiplatelet agent, NSAID, Cox-2 selective inhibitors, Corticosteroids, Selective Serotonin Reuptake Inhibitors, Anticoagulants, Gastroprotective agents, Aldosterone antagonists, Calcium channel blocker, Nitrates).

*p value < 0.05.

**p value < 0.001.

To validate whether our study conclusion could be generalized to the entire dialysis population, we re-analyzed the data in the unmatched cohort following the same study protocol (Fig. 2, Tables 2 and 3). The results of overall IR (HD vs. PD: 65.25 vs. 23.30 per 1000 patient-years, *p* < 0.001), CIR (HD vs. PD: 0.3788 vs 0.3240, *p* < 0.0001) and aSHR (1.23, 95% CI 1.15–1.33) all led to the same conclusion with greater absolute difference of values.

### Subgroup analysis stratified based on the selected comorbidities and medications

**Figure 3:**
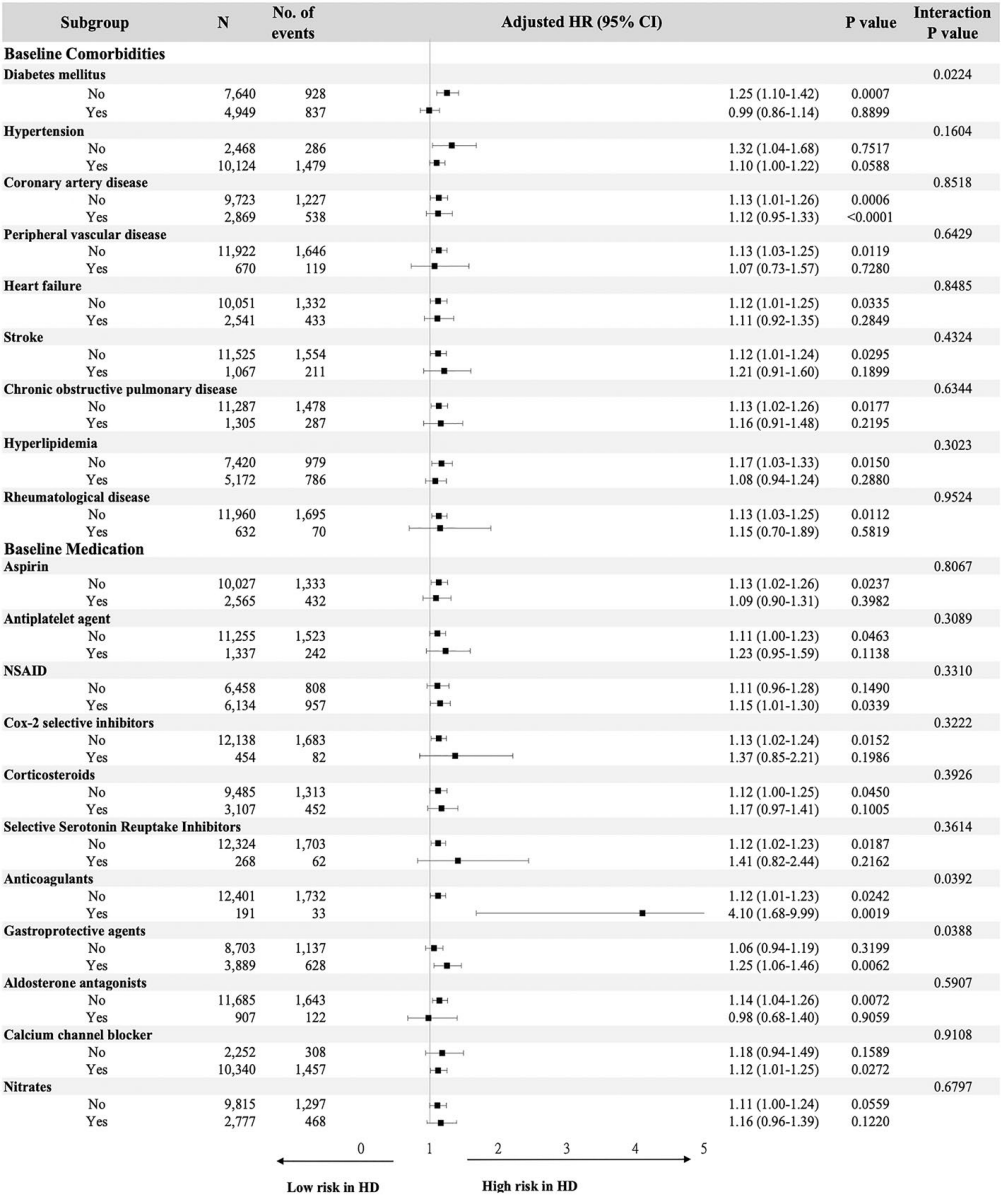
Stratified analysis of risk for gastrointestinal bleeding in matched patients undergoing hemodialysis and peritoneal dialysis using a multivariable subdistribution hazard model.

Figure 3 showed the results of the stratified analysis based on selected covariates when testing for interactions. As compared to their matched PD patients, the HD patients tended to be associated with a higher risk of GI bleeding across most of the subgroup analyses. The results of the interaction testing revealed that there were statistically significant differences in the diabetes and the use of anticoagulants and gastroprotective agents groups, which indicated that these three risk factors were modifiers of the effects of the associations of dialysis modalities with GI bleeding. Therefore, it is suggested that the presence of diabetes (aSHR: 0.99, 95% CI 0.86–1.14) and absence of the use of gastroprotective agents (aSHR: 1.06, 95% CI 0.94–1.19) will lead HD to confer a similar risk of GI bleeding as PD, while the use of anticoagulants will induce a much higher bleeding risk in HD (aSHR: 4.10, 95% CI 1.68–9.99).

## Discussion

In our study, we compared the incidence of hospitalization for GI bleeding between HD and PD from the representative national dialysis cohort database. To validate our study results, we adopted three different approaches to our analysis. The application of the propensity score-matching method made it possible to construct comparable HD and PD groups and thus improve the internal validity of the study, but at the cost of reducing external validity. Thus, we also used an unmatched cohort for analysis to confirm whether the results could be generalizable to the entire dialysis population. Finally, the stratified analysis based on the selected covariates verified whether the effect of dialysis modality could be modified by some specific conditions. Our study results derived from these different approaches were all consistent and clearly demonstrated that HD was associated with a higher risk of GI bleeding than PD. The effect was more pronounced in the unmatched cohort. Therefore, we could infer that our conclusion is robust and is applicable to real-world practice.

Few studies have investigated the risk of GI bleeding in a dialysis population, especially in terms of a direct comparison of HD and PD patients. To the best of our knowledge, this study is the first specifically designed to evaluate the risk of GI bleeding in terms of the treatment modality. Since PD patient recruitment is associated with many patient-related factors including physical ability, cognitive function or the support from family or caregivers^30^, patients on PD are generally considered younger and with less comorbidities than those on HD. Therefore, the comparison between the modalities should be done with great caution, and careful adjustment or matching for the confounders is necessary. When setting hospitalization for PUB as the primary outcome, the results of Huang et al. revealed that both HD and PD patients were associated with a higher risk of PUB as compared to the matched control (HR of HD: 11.96, 95% CI 7.04–20.31; HR of PD: 3.71, 95% CI 2.00–6.87)^11^. Since they didn’t directly compare the risk of PUB based on the dialysis modality, it could not be concluded which modality was associated with a higher risk of PUB. Another study evaluated the risk of common GI diseases in patients with and without kidney failure^21^. The results suggested that the risk of either PUB or lower GI diverticula and bleeding was significantly higher in HD patients than in PD patients (the HRs of PD were around 0.77–0.78). A third study conducted by the USRDS^20^, which evaluated the risk of GI bleeding in HD and PD patients, revealed that HD is associated with a higher risk of overall GI bleeding (PD vs. HD HR: 0.93, 95% CI 0.90–0.96), especially lower GI bleeding (PD vs. HD HR: 0.82, 95% CI 0.78–0.87). Nevertheless, the three studies referenced above were confounded by an imbalance in the baseline characteristics even after the matching process and did not include as many potential confounders as possible for adjustment^11,20,21^. Consequently, our estimated results may be closer to the true difference in the risk between HD and PD.

Several potential mechanisms may explain the higher GI bleeding risks in HD patients. First, the use of unfractionated heparin during the HD procedure or heparin locks within the central catheter vascular access will result in increases in the circulating heparin levels in HD patients^31–33^. This could further lead to bleeding events in patients with pre-existing peptic ulcers or occult GI bleeding. In addition, this hypothesis of bleeding events caused by circulating heparin is compatible with our finding in the subgroup analysis, that HD patients taking anticoagulants carry an even more remarkable increase in GI bleeding risk (Fig. 3). This finding is particularly important in clinical practice because dialysis patients often have risk factors for thromboembolic events, such as Af. According to a national cohort study in Denmark, 78.6% of dialysis patients with Af have CHA2DS2-VASc scores ≥ 2, which warrant the initiation of anticoagulant therapy^7^. However, only around 50% of dialysis patients with Af received either warfarin or direct oral anticoagulant therapy^34^, indicating that clinicians typically hesitate initiating or maintaining the use of anticoagulants in the dialysis population due to tendency toward high bleeding. Since our study and others have suggested that HD is associated with a higher bleeding risk than PD, especially in those receiving anticoagulants^19^, PD could be considered in patients on anticoagulants, and more intensive monitoring of GI bleeding in HD patients is suggested.

Second, exposure of the blood to the dialyzer membrane and tubes during the HD procedure may result in platelet activation, thrombosis, platelet dysfunction, and complement activation-induced thrombocytopenia^35–37^. Third, gastric mucosa hypoperfusion due to excessive ultrafiltration and intradialytic hypotension during HD may contribute to ischemia of the gastric mucosa and ulcer formation^38,39^. On the other hand, animal experiments have shown that PD solutions increase blood flow to the mesentery, peritoneum and omentum, without significant alterations in blood flow to the end-organs^40^. This may illustrate the different risks of GI bleeding in the dialysis modalities. Fourth, despite that both PD and HD patients have aggravated oxidative stress generated by uremia, a recent review indicated that HD patients manifest higher pro‐oxidant levels and less preservation of antioxidant molecules than PD patients do^41,42^. Since overproduction of reactive oxygen species (ROS) along with an impairment of antioxidants can lead to mucosal damage, this could increase the risk of ulcer formation and may lead to GI bleeding events^43^.

Last but not least, it has been proven that dialysis modalities have differential risks on newly developed cardiovascular events, for which anticoagulation or antiplatelet therapy might be initiated. For example, several large dialysis cohort studies showed that patients on HD carry a higher risk of developing incident coronary artery disease^44^, AMI^45^, and Af^46^ than those on PD. Therefore, HD patients are more likely to be exposed to antiplatelet agents and anticoagulants after the initiation of renal replacement therapy, and the subsequent incidence of GI bleeding is thus increased.

In contrast to intuitive conjecture, the differences in the risk of GI bleeding between HD and PD was observed among the patients taking gastroprotective agents, but not among those who were not using such agents (Fig. 3). Since patients not using gastroprotective agents should have less subjective GI symptoms and/or underlying GI lesions, this could limit the presentation of the bleeding tendency induced by HD. On the other hand, differences in bleeding risks based on HD and PD did not appear to be significant in dialysis patients with diabetes. This may be because diabetes is also associated with a high risk of bleeding events in these patients and may attenuate the risk attributable to dialysis modality. Though diabetes is known to cause thrombosis due to platelet dysfunction and hypercoagulation^47,48^, it has also been associated with high bleeding rates in several scenarios, such as in patients with pulmonary embolism on anticoagulation therapy^49^ or those who underwent percutaneous renal biopsy^50^. The cause of possible bleeding tendency among our patients, who have co-existing diabetes and renal failure, is still unclear, and further investigation is warranted.

The major strength of this study is that it is a nationwide population-based cohort study. Since the NHI program is characterized by universal coverage, high adherence and utilization, potential selection and information bias is minimized. The large sample size in the NHIRD made it possible to find enough matched pairs when performing the matching process or evaluating effects across different patient subgroups. And the results derived from the NHI, which comprises data from multiple centers, should be more representative than study results derived from a single center.

Several limitations should be addressed in this study. First, this study is a retrospective observational design. Therefore, a direct causal relationship could not be demonstrated. Second, not all potential confounders are recorded in the NHIRD, such as obesity, smoking, *H. pylori* infection, and use of self-paid prescription medications and over-the-counter medications. We did adjust for diabetes, cardiovascular diseases, stroke, and chronic obstructive pulmonary disease, which could serve as surrogate markers of obesity and smoking in the Cox models. According to previous studies, dialysis patients have a lower risk of *H. pylori* infection comparing to the general population^51^, but whether the prevalence of *H. pylori* differ between HD and PD patients is still unknown due to limited data in the PD population. Although the information of *H. pylori* infection is lacking in this study, our results revealed HD group carried a higher risk on developing both LGIB and unspecified GI bleeds (Table 3), which might indicate the lack of *H. pylori* infection had limited estimation bias on our study results. Furthermore, most of the medications listed in Table 1 that might be related to GI bleeding are prescription medications which will be recorded in the NHI database unless patients buy these medications by themselves. Only few of them, such as ibuprofen and certain painkillers that contain aspirin, can be supplied directly by a pharmacist. Due to the affordability of NHI medical services, there is a limited need for patients to purchase medications from the pharmacies. Therefore, any potential bias should be limited. Third, comorbidities and medication usage may have changed over the observation period. The use of a time-dependent variable may help to delineate the effect of interested variables on the outcome, but it might also increase the risk of adjusting for the mediators in the time-dependent Cox models analysis and finally increase the potential estimation bias^52^. By using patients’ baseline characteristics, on which the choices of dialysis modality were based in clinical settings, in our Cox models, clinicians may incorporate our study results in the process of shared decision-making with incident kidney failure patients. Fourth, we only identified GI bleeding events that led to hospitalization, thus the events of minor GI bleeding managed in the outpatient clinics were not detected, and the results could not be generalized to minor GI bleeding events. If patients were diagnosed with minor GI bleeding, they will have a high probability of receiving gastroprotective agents. Therefore, the application of gastroprotective agents can serve as a surrogate marker for minor GI bleeding. We thus included gastroprotective agents in our propensity score model construction so that its distribution was balanced in the HD and PD population after performing matching, thereby reducing the potential estimation bias in our study.

In conclusion, our study demonstrated an increased risk of newly-diagnosed GI bleeding in HD patients as compared to matched PD patients. Our findings may facilitate better decision-making in terms of selecting a dialysis modality or screening strategies for individuals at risk for GI bleeding, which will in turn further reduce the healthcare burden in the dialysis population.

## Supplementary Information

Supplementary Information.

## Data Availability

The data that support the findings of this study are available from the Ministry of Health and Welfare, R.O.C.. Restrictions apply to the availability of these data, which were used under license for this study. Data are available from Health and Welfare Data Science Center [https://dep.mohw.gov.tw/dos/cp-5119-59201-113.html] with the permission of Ministry of Health and Welfare, R.O.C.
