# Supplementary material for: Effect of dialysis modalities on risk of hospitalization for gastrointestinal bleeding
Source: Sci Rep. 2023 Jan 2;13:52. doi: 10.1038/s41598-022-26476-5 (PMC9807582; doi:10.1038/s41598-022-26476-5)
Supplement: Supplementary file 1 — Supplementary Information. [file 41598_2022_26476_MOESM1_ESM.docx]

**Supplementary Table S1.** **International Classification of Diseases, 9th Revision, Clinical Modification (ICD-9-CM) codes used to identify gastrointestinal bleeding**

| Categories | Diagnosis | ICD-9-CM code |
| --- | --- | --- |
| Upper GI bleeding |  |  |
|  | Acute gastric ulcer | 531.0x, 531.2x, |
|  | Chronic or unspecified gastric ulcer | 531.4x, 531.6x |
|  | Acute duodenal ulcer | 532.0x , 532.2x |
|  | Chronic or unspecified duodenal ulcer | 532.4x, 532.6x |
|  | Acute peptic ulcer | 533.0x, 533.2x |
|  | Chronic or unspecified peptic ulcer | 533.4x, 533.6x |
| Lower GI bleeding |  |  |
|  | Diverticulosis of small intestine with hemorrhage | 562.02 |
|  | Diverticulitis of small intestine with hemorrhage | 562.03 |
|  | Diverticulosis of colon with hemorrhage | 562.12 |
|  | Diverticulitis of colon with hemorrhage | 562.13 |
|  | Hemorrhage of rectum and anus | 569.3 |
|  | Angiodysplasia of intestine with hemorrhage | 569.85 |
|  | Hemorrhagic Dieulafoy lesion of intestine | 569.86 |
| Unspecified origin |  |  |
|  | Unspecified GI bleeding | 578.9 |
|  | Blood in stool | 578.1, 792.1 |

**Supplementary Table S2.** **International Classification of Diseases, 9th Revision, Clinical Modification (ICD-9-CM) codes used to identify comorbidities**

| Diagnosis | ICD-9-CM code |
| --- | --- |
| Diabetes mellitus | 250, 357.2x, 362.0x, 366.41 |
| Hypertension | 401-402, 405, A codes 260 and 269 |
| Coronary artery disease | 410-414 |
| Peripheral vascular disease | 440–444, 447.1 |
| Heart failure | 398.91, 425, 428, 402.x1, 404.x1, 404.x3 |
| Stroke |  |
| Ischemic | 433, 434, 436 |
| Hemorrhagic | 430, 431, 432 |
| COPD | 491-494, 496 |
| Hyperlipidemia | 272.0x–272.4x |
| Autoimmune diseases | 710, 714 |
| Malignancy | 140–208, 230–234, V10 |

**Supplementary Table S3. Anatomical Therapeutic Chemical classification system of the World Health Organization (ATC codes) used to identify baseline medications**

| Baseline Medication | ATC codes |
| --- | --- |
| Aspirin and derivatives |  |
| acetylsalicylic acid | B01AC06, N02BA01 |
| carbasalate calcium | N02BA15 |
| Other antiplatelet agent | B01AC, excluding B01AC06 |
| NSAID | M01AB, M01AC, M01AE, M01AG, M01AX |
| Cox-2 selective inhibitors | M01AH01-05 |
| Glucocorticoids | H02AB01- 11, H02AB13 |
| Selective Serotonin Reuptake Inhibitors | N06AB |
| Anticoagulants |  |
| Vitamin K antagonists | B01AA |
| Heparin group | B01AB |
| Gastroprotective agents |  |
| H2-receptor antagonists | A02BA |
| Proton pump inhibitors | A02BC |
| Misoprostol | A02BB01 |
| Aldosterone antagonists | C03DA01-04 |
| Calcium channel blocker |  |
| DHP CCB | C08CA |
| Non-DHP CCB | C08CX01, C08DA01, C08DA02, C08DB01 , C08EA01, C08EA02, C08EX01, C08EX02 |
| Nitrates | C01DA02, C01DA04, C01DA05, C01DA07, C01DA08, C01DA09, C01DA13, C01DA14 |
